# Characterization and development of EST-derived SSR markers in cultivated sweetpotato (*Ipomoea batatas*)

**DOI:** 10.1186/1471-2229-11-139

**Published:** 2011-10-20

**Authors:** Zhangying Wang, Jun Li, Zhongxia Luo, Lifei Huang, Xinliang Chen, Boping Fang, Yujun Li, Jingyi Chen, Xiongjian Zhang

**Affiliations:** 1 Crops Research Institute, Guangdong Academy of Agricultural Sciences, Guangzhou, 510640 China; 2 College of Life Science, China West Normal University, Nanchong, 637002 China

## Abstract

**Background:**

Currently there exists a limited availability of genetic marker resources in sweetpotato (*Ipomoea batatas*), which is hindering genetic research in this species. It is necessary to develop more molecular markers for potential use in sweetpotato genetic research. With the newly developed next generation sequencing technology, large amount of transcribed sequences of sweetpotato have been generated and are available for identifying SSR markers by data mining.

**Results:**

In this study, we investigated 181,615 ESTs for the identification and development of SSR markers. In total, 8,294 SSRs were identified from 7,163 SSR-containing unique ESTs. On an average, one SSR was found per 7.1 kb of EST sequence with tri-nucleotide motifs (42.9%) being the most abundant followed by di- (41.2%), tetra- (9.2%), penta- (3.7%) and hexa-nucleotide (3.1%) repeat types. The top five motifs included AG/CT (26.9%), AAG/CTT (13.5%), AT/TA (10.6%), CCG/CGG (5.8%) and AAT/ATT (4.5%). After removing possible duplicate of published EST-SSRs of sweetpotato, a total of non-repeat 7,958 SSR motifs were identified. Based on these SSR-containing sequences, 1,060 pairs of high-quality SSR primers were designed and used for validation of the amplification and assessment of the polymorphism between two parents of one mapping population (E Shu 3 Hao and Guang 2k-30) and eight accessions of cultivated sweetpotatoes. The results showed that 816 primer pairs could yield reproducible and strong amplification products, of which 195 (23.9%) and 342 (41.9%) primer pairs exhibited polymorphism between E Shu 3 Hao and Guang 2k-30 and among the 8 cultivated sweetpotatoes, respectively.

**Conclusion:**

This study gives an insight into the frequency, type and distribution of sweetpotato EST-SSRs and demonstrates successful development of EST-SSR markers in cultivated sweetpotato. These EST-SSR markers could enrich the current resource of molecular markers for the sweetpotato community and would be useful for qualitative and quantitative trait mapping, marker-assisted selection, evolution and genetic diversity studies in cultivated sweetpotato and related *Ipomoea *species.

## Background

Sweetpotato (*Ipomoea batatas*) is a hexaploid (2n = 6x = 90) dicot and belongs to the family of *Convolvulaceae*. Due to its high yielding potential and adaptability under a wide range of environmental conditions, sweetpotato is one of the world's important food crops, especially in developing countries. According to the Food and Agriculture Organization (FAO) statistics, world production of sweetpotato in 2008 was more than 110 million tons, and almost 80% came from China, with a production of around 85 million tons from about 3.7 million hectares [1]. Now sweetpotato is usually used as staple food, animal feed, industrial material and potential raw material for alcohol production. In addition, the high *beta *carotene content of orange-fleshed sweetpotato plays a crucial role to prevent vitamin A deficiency-related blindness and maternal mortality in many developing countries.

Despite its importance, sweetpotato breeding is constrained by the complexity of the genetics of this hexaploid crop and by the lack of genomic resources. Molecular markers have great potential to speed up the process of developing improved cultivars. Although several sweetpotato genetic maps have been published [2-4], the existing maps do not have sufficient markers to be highly useful for genetic studies. Thus, there is a great need for development of novel markers. With the newly developed high-throughput next generation sequencing technology, a large number of transcribed sequences have been generated for model species as well as economically important non-model plants. In addition to providing an effective approach for gene discovery and transcript profile characterization, these ESTs can be used as a cost-effective, valuable source for molecular marker development, such as single nucleotide polymorphism (SNP) and simple sequence repeats (SSRs).

DNA simple sequence repeats, also known as microsatellites, are tandem repeats of 2-6 bp DNA core sequences, which are widely distributed in both non-coding and transcribed sequences, commonly known as genomic-SSRs and EST-SSRs [5]. With the advantages of being PCR-based, reliable, co-dominant, multi-allelic, chromosome specific, and highly informative, SSRs are useful for many applications in plant genetics and breeding such as construction of high-density linkage maps, genetic diversity analysis, cultivar identification, and marker-assisted selection. Although genomic SSRs are highly polymorphic and widely distributed throughout the genome [6,7], and advances in techniques to enrich SSRs have also resulted in the accelerated development of large numbers of genomic SSR markers in many plants [8-14], it is still expensive, labor-intensive and time-consuming to develop genomic SSR markers. In contrast, EST-SSRs can be rapidly developed from EST database at lower cost. Moreover, due to their association with coding sequences, EST-SSRs can also lead to the direct gene tagging for QTL mapping of agronomically important traits and increase the efficiency of marker-assisted selection [15]. In addition, EST-SSRs show a higher level of transferability to closely related species than genomic SSR markers [13,16-18] and can be served as anchor markers for comparative mapping and evolutionary studies [19,20].

In sweetpotato, the genomic SSRs were originally developed by Jarret and Bowen [21] and used in inheritance evaluation and mutation mechanisms of microsatellite markers [22], paternity analysis [23] and assessment of genetic diversity and relationship [24,25] in cultivated sweetpotato and wild species. Later, Hu et al [26] developed 79 primer pairs from small-insert and enriched library, 27 of which showed length polymorphism among 20 sweetpotato accessions examined. At the same time, they also identified and designed 151 primer pairs from a published EST database, and 75 loci showed length polymorphism among 12 sweetpotato genotypes. Recently, large amount of ESTs were generated using pyrosequencing and Illumina paired end sequencing and provided the opportunity to develop more useful EST-SSRs for sweetpotato [27,28]. In this study, in order to reduce redundancy we combined and reassembled all these available sequences and screened a large scale of ESTs (181,615) with the objectives: (1) to analyze the frequency and distribution of SSRs in transcribed regions of cultivated sweetpotato genome; (2) to design new PCR primer pairs from these assembled sequences for sweetpotato; (3) to validate and evaluate the designed SSR primer pairs in various cultivated sweetpotato genotypes.

## Results

### Frequency and distribution of EST-derived SSR markers in sweetpotato

A total of 181,615 ESTs with an average length of 548 bp were used to evaluate the presence of SSR motifs. In order to eliminate redundant sequences and improve the sequence quality, the TIGR Gene Indices Clustering Tools (TGICL) [29] was used to obtain consensus sequences from overlapping clusters of ESTs. Assembly criteria included a 50 bp minimum match, 95% minimum identity in the overlap region and 20 bp maximum unmatched overhangs. In total, 87,492 potential unique ESTs including 28,885 contigs and 58,607 singletons were generated. For annotation of these assembled ESTs, similarity search was conducted against the UniProt database http://www.uniprot.org using BLASTx algorithm with an *E *value threshold of 10^-5^. The results showed that out of 87,492 ESTs, 53,622 (61.3%) showed significant similarity to known proteins and matched 30,924 unique protein accessions. As shown in Table 1 using the MISA Perl script http://pgrc.ipk-gatersleben.de/misa/, a total of 8,294 SSRs were identified from 7,163 unique ESTs, with an average of one SSR per 7.1 kb. Of these, 949 ESTs contained more than one SSR, and 539 were compound SSRs that have more than one repeat type. In order to identify the putative function of genes containing the SSR loci, the 7,163 EST sequences were also searched against UniProt database with *E*-value cutoff less than 10^-5^. Among of them, 4,911 had BLAST hits to known proteins in this UniProt database.

**Table 1:** Summary of EST-SSR searching results

Searching Items	Numbers
Total number of sequences examined	87,492
Total size of examined sequences (bp)	58,678,639
Total number of identified SSRs	8,294
Number of SSR containing sequences	7,163
Number of sequences containing more than 1 SSR	949
Number of SSRs present in compound formation	539
Di-nucleotide	3,413
Tri-nucleotide	3,554
Tetra-nucleotide	762
Penta-nucleotide	311
Hexa-nucleotide	254

**Figure 1:**
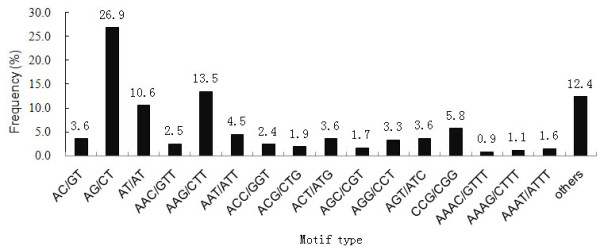
**Frequency distribution of EST-derived SSRs of sweetpotato based on motif sequence types**. X-axis is motif sequence types, and Y-axis represents the frequency of SSRs of a given motif sequence type.

The compilation of all SSRs revealed that the proportion of SSR unit sizes was not evenly distributed. Among the 8,294 SSRs, the tri-and di-nucleotide repeat motifs were the most abundant types (3,554, 42.85%; 3,413, 41.15%, respectively), followed by tetra- (762, 9.19%), penta- (311, 3.75%) and hexa-nucleotide (254, 3.06%) repeat motifs (Table 1). As shown in Table 2. SSR length was mostly distributed from 12 to 20 bp, accounting for 84.6% of total SSRs, followed by 21-30 bp length range (1,198 SSRs, 14.4%). A maximum of 94 bp di-nucleotide repeat (AG/CT) was observed. In addition, a total of 224 SSR motifs were identified, of which, di-, tri-, tetra-, penta- and hexa-nucleotide repeat had 4, 10, 31, 67 and 112 types, respectively. The AG/CT di-nucleotide repeat was the most abundant motif detected in our EST-SSRs (2,229, 26.9%), followed by the motif AAG/CTT (1,117, 13.5%), AT/TA (880, 10.6%), CCG/CGG (477, 5.8%), AAT/ATT (375, 4.5%), AGT/ATC (301, 3.6%), AC/GT (300, 3.6%), ACT/ATG (300, 3.6%), AGG/CCT (276, 3.3%) and AAC/GTT (207, 2.5%). The frequency of remaining 214 types of motifs accounted for 22.0% (Figure 1).

**Table 2:** Length distribution of EST-SSRs based on the number of repeat units

Repeat number	Di-	Tri-	Tetra-	Penta-	Hexa-	Total
4	0	0	527	248	207	982
5	0	2,188	158	50	34	2,430
6	1,286	840	48	11	9	2,194
7	788	306	18	1	2	1,115
8	468	126	6	1	0	601
9	319	50	2	0	1	372
10	193	15	0	0	1	209
11	121	12	1	0	0	134
12	110	7	1	0	0	118
13	49	5	1	0	0	55
14	17	1	0	0	0	18
≥15	62	4	0	0	0	66

### Primer design and evaluation of EST-SSR markers in cultivated sweetpotato

**Figure 2:**
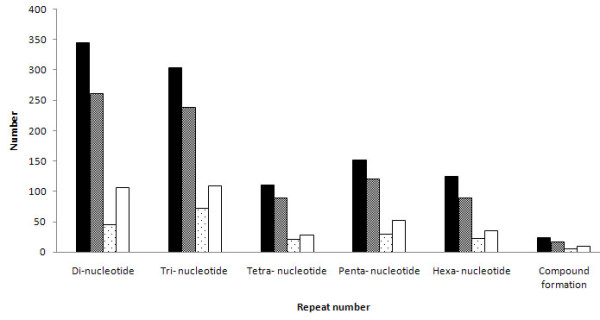
**Number of designed primer pairs and polymorphic primer pairs**. Number of primer pairs designed (black columns), primer pairs amplified (gray columns), polymorphic loci in two parents of our mapping population (dotted white columns) and polymorphic loci in the eight diverse sweetpotato cultivars (white columns).

**Figure 3:**
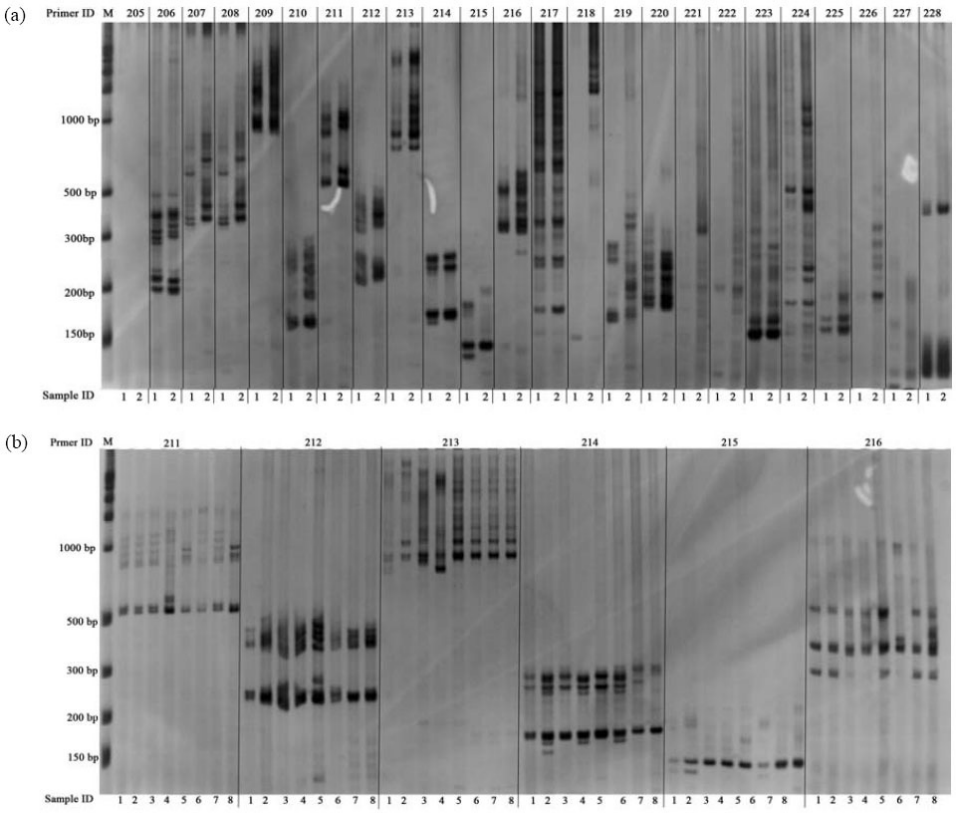
**Examples of PCR products amplified by SSR primer pairs**. (a) PCR products amplified by 24 primer pairs (GDAAS 205-228 listed on the top of the gel image) in E Shu 3 Hao (gel lanes 1 labeled in each SSR primer pair panel below the bottom of the gel image) and Guang 2K-30 (lanes 2). (b) PCR products in eight sweetpotato cultivars amplified by six effective SSR primer pairs selected from figure 3a. Within each primer pair image panel, the order of DNA samples from left to right is NANCY HALL (lanes 1), Sheng Li Bai Hao (lanes 2), AB940078-1 (lanes 3), Nortnnigo (lanes 4), Tai Nong 57 (lanes 5), Hua Bei 553 (lanes 6), Yu Bei Bai (lanes 7), and Bao Ting Zhong (lanes 8). Standard size markers are given on left side.

EST-SSRs of sweetpotato have been developed previously [26-28]. In order to ensure designing of novel EST-SSR primer pairs only, the primers from these published microsatellites were compared against the 7,163 potential unique SSR-containing sequences. A total of non-repeat 7,958 SSR motifs were identified in this study. Based on these SSR-containing sequences, 1,060 pairs of high-quality SSR primers were designed using Primer Premier 6.0 (PREMIER Biosoft International, Palo Alto CA). Of these designed primers, 345, 303, 111, 152, 125 and 24 were for di-, tri-, tetra-, pena-, hexa-nucleotide repeats and compound formation repeats, respectively (Figure 2). After being tested in E Shu 3 Hao and Guang 2K-30, 897 primer pairs (84.6%) were successfully amplified. The remaining 163 primers failed to generate PCR products at various annealing temperatures and Mg^2+ ^concentrations and would be excluded from further analysis. Of the 897 working primer pairs, 811 amplified PCR products at the expected sizes, and 65 primer pairs resulted in larger PCR products than what expected, and PCR products of the other 21 primer pairs were smaller than expected. The 897 primers were employed for further validation in eight diverse sweetpotato cultivars, and 816 could generate clean and reproducible amplicons in the eight cultivars. Examples of PCR products amplified by SSR primer pairs in E Shu 3 Hao and Guang 2K-30 and in the eight cultivars were shown in Figure 3a, b. Marker names for the 816 primer pairs, along with SSR motif, primer sequences, SSR containing sequences, Tm (melting temperature), expected product length are provided in the additional files (Additional file 1 Table S1).

### Polymorphism of EST-derived SSR markers in cultivated sweetpotato

The polymorphism assessment was first examined in E Shu 3 Hao and Guang 2K-30. Among the 816 effective SSR primer pairs, 195 (23.9%) were polymorphic between the two mapping parents. A total of 644 alleles at polymorphic loci were detected and the average number of alleles per SSR marker was 3.30 with a range of 2-10, based on the dominant scoring of the SSR bands characterized by the presence or absence of a particular band (Additional File 1). Polymorphisms could be observed for 45 di-, 72 tri-, 21 tetra-, 29 penta-, 22 hexa-nucleotide repeats and 6 compound formation repeats (Figure 2). The results of a BLASTx search showed that 68.7% (134) of the polymorphic SSR loci could be associated with known or uncharacterized functional genes.

The polymorphism of the 816 EST-derived SSRs was further evaluated in eight diverse accessions of cultivated sweetpotatoes. The results showed that 342 (41.9%) primers were polymorphic, with a total of 1,004 alleles detected (Additional file 1). The average number of alleles per locus was 2.94 with a range of 2-11 alleles. A maximum of 11 alleles was observed for primer GDAAS1073. The PIC values varied from 0.22 to 0.88 with an average value of 0.35. Polymorphisms could be observed for 106 di-, 109 tri-, 28 tetra-, 53 penta-, 36 hexa-nucleotide repeats and 10 compound formation repeats (Figure 2). Among of these 342 polymorphic SSR loci, 266 had BLAST hits to known proteins in the UniProt database.

## Discussion

### Frequency and distribution of sweetpotato EST-SSRs

The frequency of SSRs in SSR containing ESTs can accurately reflect the density of SSRs in the transcribed region of the genome. Using Sanger and next generation sequencing, a large number of EST sequences for sweetpotato have been generated. These sequences offered us an opportunity to discover novel genes, also provided a resource to develop markers. However, there is abundant redundancy in these EST sequences due to the non-normalized cDNA libraries and submission by different researchers. In this study, in order to reduce the redundancy and avoid overestimation of the EST-SSR frequency, SSR searching was performed following redundancy elimination. A total of 87,492 potential unique EST sequences (about 58.7 Mb) were used for SSR searching and 7,163 ESTs (8.2%) contained SSR motifs, generating 8,294 unique SSRs. The result of SSR abundance was in agreement with the report by Hu et al (9.1%) [26]. These two results indicated that the abundance of SSRs for sweetpotato ESTs was relatively higher than that for other species, e.g. peanut (6.8%) [30], barley (3.4%), maize (1.4%), rice (4.7%), soyghum (3.6%), wheat (3.2%) [31], *Medicago truncatula *(3.0%) [17], *Epimudium sagittatum *(3.4%) [32]. In this work, the frequency of occurrence for EST-SSRs was one EST-SSR in every 7.1 kb. In previous reports, an EST-SSR occurs every 13.8 kb in *Arabidopsis thaliana*, 3.4 kb in rice, 8.1 kb in maize, 7.4 kb in soybean, 11.1 kb in tomato, 20.0 kb in cotton and 14.0 kb in poplar [33]. However, a direct comparison of abundance estimation and frequency occurrence of SSR in different reports is difficult due to the fact that the estimates were dependent on the SSR search criteria, the size of the dataset, the database-mining tools and the EST sequence redundancy.

In earlier reports, tri-nucleotide repeats were generally the most common motif found in both monocots [19] and dicots [17]. In the present investigation, tri-nucleotide repeat was also found to be the most abundant SSRs, followed by di-, tetra-, penta, and hexa-nucleotide (Table 1). As shown in Figure 1, the most dominant di- and tri-nucleotide motif types were AG/CT (26.9%) and AAG/CTT (13.5%), respectively. These were in agreement with recent studies in cultivated peanut (*Arachis hypogaea *L.) [30], *Epimudium sagittatum *[32], and many dicotyledonous species [34]. The previous studies of arabidopsis [33] and soybean [35] also suggested that the tri-nucleotide AAG motif may be common motif in dicots. In contrast, the most frequent tri-nucleotide repeat motifs were (AAC/TTG)n in wheat, (AGG/TCC)n in rice, and (CCG/GGC)n in maize, barley and sorghum [31,36,37]. The abundance of the tri-nucleotide CCG repeat motif was favored overwhelmingly in cereal species [31,36,38] and also considered as a specific feature of monocot genome, which may be due to the high GC content and consequent codon usage bias [5,39]. But interestingly, in this study, the second most dominant tri-nucleotide repeat motif was CCG/CGG (5.8%), following AAG/CTT. This result was similar to the previous report in sweetpotao [26], which also showed CCG repeat was one of high abundant tri-nucleotide motifs.

### Validation and polymorphism of sweetpotato EST-SSRs

In this study, in order to remove possible duplicate of published EST-SSRs of sweetpotato, the primers from the published EST-SSR markers were compared against the 7,163 unique SSR-containing sequences. A total of 336 pairs of SSR primers were found matching to SSR-containing sequences of this investigation, and the matched sequences were excluded from primer designing later. Among the 336 SSR primers, seven pairs of primers designed by Wang *et al*. [27] and seven primer pairs (six designed by Schafleitner *et al*. [28] and one by Hu *et al*. [26]) matched to the same 7 SSR-containing sequences (Table 3). This indicates that the seven SSR primer pairs amplify the same SSR loci as the other seven SSR markers.

**Table 3:** Repeated SSR loci among published SSR markers in sweetpotato

**SSR ID of Wang et al**.	**SSR ID of Hu et al**. **and Schafleitner et al**.	SSR sequence ID in this study	Similarity (%)	Annotation
PP45/46	BU691547	IBGI_19821singleton	100	hypothetical protein
PP65/66	IBS25	IBGI_1430Contig2	100	At2g03070 [Arabidopsis thaliana]
PP85/86	IBS153	IBGI_11291Contig1	100	no hit
PP115/116	IBS80	IBGI_21250Contig1	100	Transcription factor Myb n = 1
PP119/120	IBS94	IBGI_19573Contig1	100	no hit
PP151/152	IBS204	IBGI_2434Contig3	100	no hit
PP165/166	IBS88	IBGI_12324Contig1	100	no hit

Based on these non-repeat SSR-containing sequences, a total of 1,060 primer pairs were designed and used for validation of the EST-SSR markers in sweetpotato. Of these, 897 primer pairs (84.6%) yielded amplicons in the two parents of our mapping population. This result was similar to EST-SSR amplification rate in sweetpotato [26,28] and many other studies in which a success rate of 60-90% amplification has also been reported [37,40-43]. In those studies (except [26]), they also reported a similar success rate of amplification for both genomic SSRs and EST-SSRs. However, in sweetpotato, the amplification efficiency of EST-SSRs was much higher than that of genomic SSRs [22,26]. The higher efficiency of PCR amplification of EST-SSRs may be attributed to the reason that sequence data for primer design were from relatively highly conserved transcribed regions, not randomly from total genomic libraries. Just due to the reason that EST-SSRs were from highly conserved transcribed regions, they were reported to be less polymorphic, but have higher transferability and better applicability than genomic SSR markers in crop plants [44-47]. The 816 amplifiable EST-SSR primers will further be used for validation of the amplification and assessment of the polymorphism among wild *Ipomoea *species.

As is commonly known, polymorphic SSR markers are important for research involving genetic diversity, relatedness, evolution, linkage mapping, comparative genomics, and gene-based association studies. In the present investigation, SSR primer polymorphism was first examined in the two parents of our mapping population. Among the tested primers, 195 were polymorphic between the two mapping parents. These markers would be useful for construction of an SSR-based linkage map. Furthermore, among of these working primer pairs, 342 (41.9%) showed polymorphism in the eight cultivated sweetpotatoes. This value was lower than earlier studies, in which 62.5% and 67.2% SSRs revealed to be polymorphism in different test set [26,28]. A small number of DNA samples and DNA samples from a different geographic origin may result in a different polymorphism. For example, a relatively high level of polymorphism was reported in cassava when the number of accessions used increased from 38 to over 500 [48]. Additionally, sufficient published data from other plant and animal species have proved that tri-nucleotide SSR loci possess low variability than di-nucleotide containing SSR loci [49-51]. In our results, no correlation was found between the number of nucleotide motif repeats and the level of polymorphism (as shown in Figure 2).

## Conclusion

In this study, in addition to the characterization of EST-derived SSR markers in cultivated sweetpotato, we designed and validated 1,060 SSR markers in two parents of our mapping population. Among the effective primers, 41.9% of them showed polymorphism in eight sweetpotato cultivars. These developed SSR markers will provide a valuable resource for genetic diversity, evolution, linkage mapping, comparative genomics, gene-based association studies, and marker-assisted selection in sweetpotato genetic study. Since these markers were developed based on conserved expressed sequences, they may be valuable for functional analysis of candidate genes. To the best of our knowledge, this is the first attempt to exploit EST dababase and develop large numbers of SSR markers in sweetpotato.

## Methods

### Plant materials and DNA extraction

In the present study, two parents of a mapping population, E Shu 3 Hao and Guang 2k-30, and 8 accessions of cultivated sweetpotatoes were used (Table 4). The leaf samples of each accession were collected by mixing equal amount of leaf tissues from 6 plants from National Germplasm Guangzhou Sweetpotato Nursery located in Crops Research Institute, Guangdong Academy of Agricultural Sciences, Guangzhou, China. The genomic DNA was extracted using a modified CTAB method [52]. DNA quality and quantity were measured by a Nanodrop spectrophotometer (Thermo Fisher Scientific Inc., Waltham, MA, USA) and 0.8% agarose gel electrophoresis, respectively.

**Table 4:** Sweetpotato accessions used for EST-SSR validation and evaluation

Nursery No	Cultivar name	Origin	Description
GN1284	E Shu 3 Hao	China	Improved variety, mapping parent
GN1337	Guang 2K-30	China	Improved variety, mapping parent
GN0442	Nancy Hall	USA	Introduced variety
GN0520	Sheng Li Bai Hao	Japan	Introduced variety
GN1245	AB940078-1	Peru	Improved variety
GN0815	Nortnnigo	Philippines	Introduced variety
GN1256	Tai Nong 57	Taiwan	Improved variety
GN0386	Hua Bei 553	China	Improved variety
GN0069	Yu Bei Bai	China	Landrace
GN0010	Bao Ting Zhong	China	Landrace

### Data mining for SSR marker

A total of 181,615 EST sequences including 66,418 (31,685 contigs and 34,733 singletons) from sweetpotato gene index established by Schafleitner *et al *[28], 56,516 developed by Wang *et al *and 58,681 generated in house were used in this study. These ESTs were assembled using the TGICL program [29]. A Perl script known as MIcroSAtellite (MISA http://pgrc.ipk-gatersleben.de/misa/) was used to mine microsatellites. In this work, the search was conducted for sequences that showed at least six repetitions for di-, five repeat units for tri-, and four repetitions for tetra-, penta- and hexa-nucleotides, excluding polyA and polyT repeat. Frequency of SSR refers to kilobase pairs of EST sequences containing one SSR.

### Primer design and PCR amplification

In order to remove possible duplicate of published EST-SSRs, comparison was performed using the primers from the published 370 EST-SSR markers (75 [26], 195 [28], 100 [27]) against the 7,163 unique SSR-containing sequences. Each set of sequences was compared by specialized NCBI blast program called bl2seq using default parameters with the exception that the word size algorithmic parameter was changed from 28 to 16 due to the short length of the primers (18-24 bp) [53].

Sequences that showed the longest repetitions and flanking regions that quantified primer design were selected for PCR primer design using primer premier 6.0 (PREMIER Biosoft International, Palo Alto, CA). Primers were designed based on the following core criteria: (1) primer length ranging from 18 bp to 24 bp; (2) melting temperature (Tm) between 52°C and 63°C with 60°C as optimum; (3) PCR product size ranging from 100 to 350 bp; (4) GC% content between 40% and 60% with amplification rate larger than 80%. The parameters were modified when unsuitable primer pairs were retrieved by the program. When two distinct microsatellite sequences were present in one EST sequence at distant sites, primer pairs were designed respectively. When two loci were in close proximity in one sequence, the primer pairs were designed outside of these micorsatellites.

PCR analysis was performed in a total volume of 20 μl reaction mixture that contained 40-50 ng template DNA, 1× PCR buffer (20 mM Tris pH 9.0, 100 mM KCl, 2.0 mM MgCl_2_), 200 μM of each of the four dNTPs, 0.2 μM of each of the forward and reverse primers, and one unit of Taq DNA polymerase with the following cycling profile: 1 cycle of 5 min at 94°C, an annealing temperature of 55-65°C for 35 cycles (1 min at 94°C, 30 s at 55-65°C, 45 s at 72°C) and an additional cycle of 10 min at 72°C. Each of the primer pairs was screened twice to confirm the repeatability of the observed bands in each genotype. PCR products were separated on 6% polyacrylamide denaturing gels. The gels were silver stained for SSR bands detection.

### Primer screening, evaluation and data collection

Designed primer pairs were firstly screened using E Shu 3 Hao and Guang 2k-30 for their effectiveness to amplify SSR fragments of the expected size and to detect allele polymorphism. The effective primer pairs from the screening were confirmed and evaluated further on the following eight cultivars. Every PCR reaction was performed twice. The allelic frequencies were calculated for the samples analyzed. The genetic diversity of the samples as a whole was estimated based on the number of alleles per locus (total number of alleles/number of loci), the percentage of polymorphic loci (number of polymorphic loci/total number of loci analyzed) and polymorphism information content (PIC). The polymorphism was determined according to the presence or absence of the SSR locus. The value of PIC was calculated using the formula $\text{PIC}=1+\overset{\text{n}}{\underset{\text{i}=1}{\sum }}{\text{P}}_{\text{i}}^{2}$where P_i _is the frequency of an individual genotype generated by a given EST-SSR primer pair and summation extends over n alleles.

## Authors' contributions

ZYW conceived, organized and planned the research, and drafted the manuscript. LJ designed PCR primers and participated in DNA extraction and SSR experiment. ZXL participated in primers designing and SSR experiment. LFH participated in primer designing. XLC participated in polyacrylamide denaturing gel running. BPF participated in design and coordination. YJL participated in manuscript preparation and revision. JYC and XJZ provided the plant material for SSR analysis. All authors read and approved the final manuscript.

## Supplementary Material

Additional file 1 **Table S1-Primer sequences for EST-SSR markers in sweetpotato**. Click here for file
